# Reducing the loss of vaccines from accidental freezing in the cold chain: The experience of continuous temperature monitoring in Tunisia

**DOI:** 10.1016/j.vaccine.2014.10.080

**Published:** 2015-02-11

**Authors:** John Lloyd, Patrick Lydon, Ramzi Ouhichi, Michel Zaffran

**Affiliations:** a PATH, Ferney-Voltaire, France; b World Health Organization, Geneva, Switzerland; c World Health Organization, Tunis, Tunisia

**Keywords:** Freezing vaccine, Cold chain, Domestic refrigerator, Temperature monitoring

## Abstract

Accidental freezing of vaccines is a growing threat and a real risk for national immunization programs when the potency of many vaccines can be compromised if these are exposed to sub-zero temperatures in the cold chain. In Tunisia, this issue is compounded by using sub-standard domestic cold chain equipment instead of equipping the program with medical refrigerators designed specifically for storing vaccines and temperature sensitive pharmaceuticals. Against this backdrop, this paper presents the findings of a demonstration project conducted in Tunisia in 2012 that tested the impact of introducing several freeze prevention solutions to mitigate the risk of accidental freezing of vaccines. The main finding is that, despite the continued use of underperforming domestic refrigerators, continuous temperature monitoring using new technologies combined with other technological interventions significantly reduced the prevalence of accidental exposure to freezing temperatures. These improvements were noticed for cold chain storage at regional, district and health center levels, and during the transport legs that were part of the demonstration conducted in the regions of Kasserine in the South-Eastern part of Tunisia. Subsequent to introducing these freeze prevention solutions, the incidence of freeze alarms was reduced and the percent of time the temperatures dropped below the 2 °C recommended threshold. The incidence of freeze alarms at health center level was reduced by 40%. Lastly, the solutions implemented reduced risk of freezing during transport from 13.8% to 1.7%. Although the solution implemented is not optimal in the longer term because domestic refrigerators are used extensively in district stores and health centers, the risk of accidental freezing is significantly reduced by introducing the practice of continuous temperature monitoring as a standard. The management of the cold chain equipment was strengthened as a result which helps protect the potency of vaccines to the areas of most difficult access.

## 1 Introduction

Some immunization programs in the world may be vaccinating their population with vaccines that have lost their potency [1] due to cold rather than heat as the better-known hazard. Vaccine freezing is a known problem in many countries [2,3] and in Tunisia the need to find a solution was triggered by three key concerns. Firstly, an effective vaccine management evaluation (EVM [4]) included a small scale temperature monitoring study conducted in 10 refrigerators used to store vaccines at district and health center facilities which showed persistent challenges in the vaccine supply chain and indicated that 60% of the refrigerators had regular negative temperature excursions [5]. And while the EVM assessment revealed that refrigerator temperatures were monitored by standard thermometers twice a day as recommended, freezing temperatures were frequently occurring in certain refrigerators and for prolonged periods [6]. Secondly, Tunisia's national immunization program has been equipping its districts and health facilities with domestic refrigerators procured on the local market in preference to pre-qualified, imported cold chain equipment. Results from laboratory testing of the best performing domestic models used in the country showed that these failed to meet the norms 1 and standards set by the World Health Organization (WHO) [7]. Thirdly, as Tunisia plans to introduce newer and more expensive vaccines that are highly sensitive to freezing, a solution to prevent vaccine freezing was urgently needed.

Against this backdrop, this paper describes the findings of a demonstration that was a part of project Optimize—a WHO and PATH collaboration aimed at demonstrating transformational solutions for vaccine supply systems in low and middle income countries [8]. This demonstration in Tunisia documents the impact on accidental freezing incidence of introducing a continuous temperature monitoring system and procedure into the vaccine supply chain. The findings from this research will contribute to the evidence based on new approaches and solutions to safeguard the potency of vaccines in an end-to-end in-country vaccine supply chain.

## 2 Methods^2^

The methodology used for this research triangulated three sequential activities in a pilot region of Kasserine between the year 2011 and 2012. The first activity was to generate better evidence on the performance of the cold chain system by conducting a baseline assessment of key temperature metrics. The second activity was to implement a temperature monitoring solution in agreement with the Ministry of Health in Tunisia. The third activity was to measure the impact of the solution against the same baseline assessment temperature metrics. In the remainder of Section 2, each activity will be described in more depth.

### 2.1 Baseline assessment

The Baseline assessment took place in 2011 for transport and in January and February 2012 for storage. Temperature metrics of storage were collected using two different approaches. The first was to track temperature in a field setting and the second was to track temperature performance of the cold chain by testing various models of equipment in a laboratory setting. A specialized laboratory in Tunis conducted the tests according to WHO norms and protocols. However, due to delays in testing, the results were not available until April 2012 [9]. The 2011 temperature study in the field helped guide the selection of four refrigerator models assembled and available in the Tunisian market. The models with the least energy consumption, corresponding to recent Government regulations, and with a record of reliability in the district stores and health centers were chosen. Since all regional vaccine stores, were equipped with refrigerator models that met WHO/PQS norms for storing vaccines (WHO prequalified), Kasserine regional store equipment was not laboratory tested.

In the field, standard stem or dial thermometers are used for temperature monitoring and the practice for health workers is to check the thermometer temperatures twice daily, and manually record the temperatures on a tracking sheet. Unfortunately, for the purposes of this study, a thorough review of these temperature tracking sheets is not an effective way to gauge if inadvertent freezing temperatures are occurring in the cold chain. As a remedy, continuous recorded temperatures data was required for the analysis. As such, the baseline temperature assessment for vaccine storage was conducted for a two month period between January and February 2012. Temperature data were collected using continuous temperature recorders,^3^ configured to make readings at 15 min intervals and placed inside each refrigerator at the center of the vaccine load. A total of 43 refrigerators were monitored as follows: six refrigerators at the Kasserine regional store; one refrigerator each of the 12 district stores in Kasserine, and 25 health centers located within the three key project districts of Feriana, Foussana, and Hassi-el-Frid. For vaccine transport, the assessment took place from September to November 2011 and was planned to include two for the Optimize project districts. The transport leg between Kasserine regional store and two district stores were tracked, and subsequently the leg between the two districts and the 25 health centers. A total of 31 deliveries to the health centers were assessed. Note that the standard practice in Tunisia is to transport vaccines in domestic cooler boxes filled with frozen-water packs without any temperature monitoring devices. For the baseline assessment, the configuration of temperature recorders was reduced to a 5 min interval to ensure there are sufficient readings for the shortest of the transport journeys monitored. In the same manner as for refrigerators, the temperature recorders were placed at the center of the vaccine load inside the cooler box.

For the analysis of the baseline temperature metrics, the data from various temperature loggers were compiled and aggregated. For storage in fixed cold chain equipment, a total of 504,874 readings were made throughout the baseline reporting period. For storage in mobile cold chain equipment, a total of 516 readings were made along various transport routes, and retrieved for analysis.

### 2.2 Implementing the vaccine freeze-prevention solution

In collaboration with the Ministry of Health at national and local levels, a two-pronged approach to reduce the risk inadvertent freezing in the vaccine cold chain of Tunisia was implemented in the same facilities where the baseline assessment was conducted. The demonstration was conducted for a 6-month period between March and September 2012. The first approach was to introduce the technology and practice of continuous temperature monitoring for both vaccine storage and vaccine transport procedures. The second approach was to introduce new high performance containers used to transport vaccine, and to switch to “Phase Change Material” (PCM) packs. Each of the two approaches is described in more detail below.

#### 2.2.1 Introducing continuous temperature monitoring of storage and transport

In replacement of stem or dial thermometers with verification twice daily where research shows that this approach fails to safeguard against damaging temperature excursions [10], continuous temperature recording devices were installed in the 43 refrigerators and for 30 vaccine transport journeys—a few more than were assessed in the baseline. The same baseline configuration for the temperature loggers was kept so that comparisons between the baseline and the demonstration temperatures could be made. Visual temperature alarms setting were made according to the WHO time and temperature threshold as follows: a low alarm would be triggered in the cold chain if temperatures were at −0.5 °C or less for 60 consecutive minutes [11]. When a low alarm is triggered, there is a high risk that vaccines would have lost their potency from exposure to excessive freezing temperatures. The control protocol is to conduct the “shake-test” when possible [12].

In addition to providing continuous temperature monitoring devices, Standard Operating Procedures (SOPs) were developed for all health workers that would be managing or transporting vaccines in the demonstration site. Health workers in the demonstration sites were invited to follow training the temperature monitoring devices; the procedures to follow in the event of an alarm (ex: how to log the alarms and analyze root causes); and the method for transferring the monthly temperature data back up to the national immunization program in Tunis. The data would be centralized into a national database for detailed analysis. In exchange, monthly temperature reports were sent back to each of the demonstration sites. These were discussed during supervisory visits as a way of building capacity in the continuous temperature monitoring system.

#### 2.2.2 Using innovative cold chain technologies for transport

**Fig. 1:**
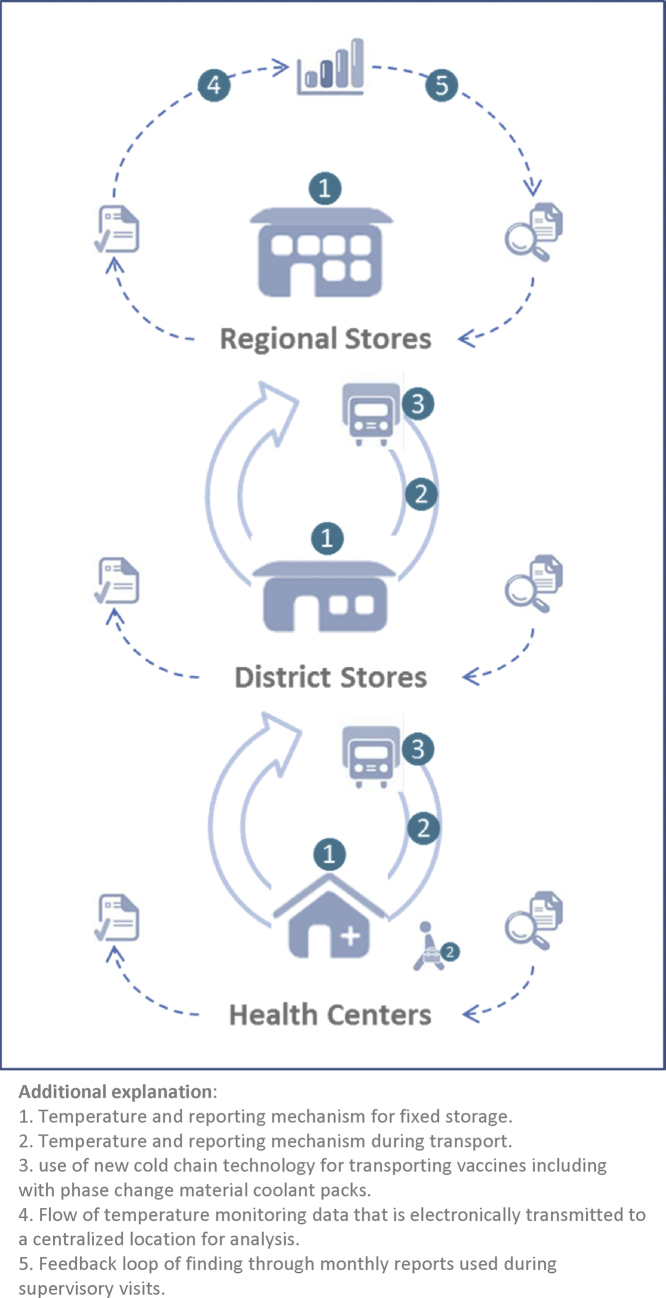
Design of the solution demonstrated.

Following the steps of distribution in (see Fig. 1), transport of vaccines in Tunisia is traditionally done in domestically produced picnic cooler boxes filled with frozen-water packs to keep the temperatures in the correct ranges. Given that compliance for conditioning ice-packs and adequately packing vaccines is limited vaccines are often placed right next to, or on-top of a frozen water pack. This practice increases the risk of vaccine freezing during transport.

For each of the demonstration sites that transport vaccines, high performance cold boxes designed for temperature sensitive health products were provided. The 15 h hold-over time of this equipment exceeds the duration of any transport journey for vaccines in the entire country. In addition, the standard frozen-water packs were replaced with coolant packs filled with Phase Change Material (PCM).^4^ The particularity of this PCM technology is that it can freeze a positive temperature of +5 °C. Packs filled with PCM provide about half of the cooling as a frozen-water pack but without the risk of freezing.

In addition to providing continuous innovative cold chain technologies for transport, standard operating procedures were developed for all health workers transporting vaccines in the demonstration sites. These health workers were invited to follow the same training course on the temperature monitoring procedures where an additional session on how to pack vaccines in these new cold boxes was organized.

### 2.3 Measuring the impact

Measuring the impact of the solution to avoid inadvertent freezing in the vaccine was an exercise in comparing the temperature monitoring metrics measured during the baseline assessment, against the monitoring of temperatures put in place as part of the continuous temperature monitoring system implemented. For this comparative analysis, key temperature indicators were generated including the number of freeze alarms; the proportion of time vaccine were kept at the recommended ranges of +2 °C to +8 °C; and the proportion of time temperature were under 2 °C. The analysis of temperature was conducted for both storage and transport.

## 3 Results

**Fig. 2:**
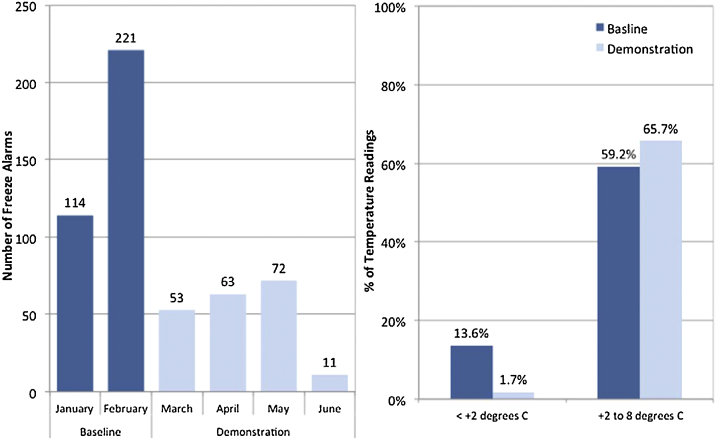
Overall impact of the vaccine freeze-prevention solution on vaccine storage at all levels.

Significant improvements were discovered (see Fig. 2 and Table 1) when comparing the temperature performance of the cold chain system before and after the implementation of the vaccine freeze-prevention solution in the demonstration sites of the regions of Kasserine in the South-Eastern part of Tunisia. These improvements were noticed for cold chain storage at regional, district and health center levels, and during the two transport legs that were part of the demonstration.

### 3.1 Risk of freezing in storage reduced

In reviewing the findings from the baseline assessment of temperature metrics (see Fig. 2), the analysis confirmed the suspicions that inadvertent freezing was occurring in storage. The temperature data indicated a cumulative total of 335 freezing alarms occurred during a two month period between January and February (the baseline for storage). These alarms occurred in 43 domestic refrigerators that were part of the demonstration project—an average of 3.9 alarms per refrigerator per month.

In spite of March being as almost as cold as February in Kasserine, this baseline frequency of alarms was reduced to a total of 199 alarms during the four months (March–June) of the project, an average of 1.2 alarms per refrigerator per month and the overall trend is downwards. The two main reasons for this dramatic reduction in alarms were that first, some facility staff were unaware of the problem when a simple solution existed (thermostat adjustment or respecting air movement spaces in the load). Second, where the facility staff were aware of a problem requiring replacement or repair, they were not able to engage the supervisors to tackle the problem of inadequate performance or to take any action.

A more in-depth analysis of the baseline data highlighted two findings. The first finding is that not all demonstration sites had freeze alarms. The extent of the problem is least pronounced at regional level and most pronounced at health center level. The better performance at regional level is due to the fact that in Tunisia this level includes WHO prequalified refrigerators. In the case of Kasserine, of the four refrigerators use at the regional store, only one is a domestic refrigerator, and the other three are WHO prequalified refrigerators for vaccines. For this reason, the temperature performance at regional level was high—no freeze alarms and only 0.5% of the time where temperatures dipped below +2 °C for two reasons. The first is a seasonality effect and where these dips occurred in the winter months were ambient temperatures in Kasserine are known to drop to freezing point, and where health facilities are poorly insulated and/or have limited heating. The second reason is that the domestic refrigerator use at the regional store is the reason why performance weakened during the demonstration period. For the health center level findings, the poor temperature performance is largely the result of the domestic refrigerators used. The second finding is that the relatively high incidence of alarms in baseline and even project data, was concentrated around certain brands of domestic refrigerators that were performing particularly poorly, and putting vaccines at risk of potency damage and loss. Cross-checking the refrigerators brands with those tested in the laboratory setting confirmed that all were inadequate for storing vaccines. While it is difficult to know how the high incidence of freezing at health center level would have affected the potency of the vaccines stored in those centers, it does beg the question of whether damaged vaccines are being used.

Although freeze alarms are one temperature metric, the proportion of time the temperatures were within the WHO recommended temperature ranges of +2 °C to +8 °C is an additional metric for measuring the risk of inadvertent or accidental exposure to freezing temperature. The analysis of this baseline temperature metrics revealed that overall, the recommended temperature ranges were maintained 73.9% of the time although this proportion would vary by level. The recommended ranges were maintained 77.8% of the time at district vaccines stores, and 63.2% of the time at health facility level during the baseline assessment period.

When comparing the baseline temperature metrics against those monitored throughout the implementation period of the demonstration, we find significant improvements the prevalence of accidental exposure to freezing temperatures was significantly reduced. Subsequent to introducing the freeze prevention solution, the number of freeze alarms dropped significantly to 116 alarms between March and April and 83 alarms between May and June. Another way to highlight the impact of demonstration is to compare the monthly average of 167 alarms during the baseline assessment against the 50 alarms per month during the demonstration phase. At district levels, freeze alarms dropped to zero during the demonstration period while freeze alarms still occurred at health center level but reduced by more than 40%. The percentage of temperature readings above +8 °C rose as a result of the use of PCM packs by 5.4% which is not a significant problem compared to the risk of freezing when the maximum deviations do not exceed +20 °C in transport.

### 3.2 Risk of freezing in transport reduced

The solutions implemented between district and health center levels reduce the risk of freezing during the transport from 13.8% during the baseline (31 deliveries) to 1.7% during the demonstration (15 deliveries). This proportion did not drop to 0% as expected. The residual 1.7% of < + 2 °C exposures were due to health worker misunderstanding of PCM conditioning procedure following training. In some instances, the health workers put the PCM packs in the freezer with tradition frozen water packs rather than in a refrigerator. As a result, the PCM packs were deep frozen and could not operate in the way they are designed. Once this practice had been addressed by supervisory visits, compliance was high and there were no more occurrences of freezing during transport.

## 4 Discussion

Tunisia, like many other middle income countries relies on cheaper and locally assembled domestic refrigerators and cold boxes for storing and transporting vaccines. As discovered by testing the better performing models in a laboratory setting, the equipment used at the district and health center levels failed to meet the minimum norms and standards set by WHO for vaccines. All models showed important performance weaknesses that clearly impact on the quality of vaccine handling, equipment lifetime and cold chain reliability. One of these performance weaknesses is not being able to maintain the recommended temperature ranges of +2 °C to 8 °C and the risk of vaccine freezing. These weaknesses of domestic refrigerators and the consequences when they are used to store vaccines are well known although few countries with refrigerator manufacture have sought a solution [13].

Given the risks, a temperature monitoring solution was implemented in Tunisia and successfully demonstrated that the use of electronic/continuous temperature monitoring and freeze prevention technologies can help significantly to reduce the incidence of inadvertent freezing of vaccine. Continuous monitoring of storage temperatures detected temperature excursions and freeze events even when health workers were not present. In addition, the ability to analyze the temperature data in real time allowed health workers to take corrective action and specific standard operating procedures were put in place to help health workers respond appropriately. In addition to protecting the potency of vaccines against freezing, the temperature monitoring solution strengthened the cold chain equipment management by detecting refrigerators that needed to be replaced or those whose thermostat needed to be adjusted—something that was not possible when simple dial thermometers were use previously.

On vaccine transport, the practice of routine inspection of temperature during distribution enabled health workers to ensure that vaccines had not been compromised by the time they arrived at destination. Temperature recording during transport further highlighted the benefits of having a high performance storage container in association with PCM coolant packs that eliminated occurrences of freezing exposure. Poor compliance with the current policy to pre-condition frozen water packs before loading the vaccine into a cold box for transport were putting vaccines at risk of freezing—particularly those closest to the un-conditioned frozen packs. Removing need for pre-conditioning with PCM packs almost entirely removes the risk of freezing during transport and has important managerial benefits from streamlining and speeds up the process of preparing vaccines deliveries (no longer the need for time-consuming pre-conditioning of frozen water packs).

While this study provides evidence on the benefits of implementing freeze prevention solutions, several limitations are noteworthy. The first is that the solution implemented for storage did not entirely mitigate all risks of freezing and highlights that continuous temperature monitoring is a necessary but not sufficient intervention to prevent vaccine freezing when domestic refrigerators are being used by the national immunization program. The second is that for transport, the cost-effectiveness of using PCM packs remains unclear. While this technology can eliminate the risk of freezing during transport, it continues to be expensive. In addition, the demonstration results showed that an additional refrigerator is needed for cooling PCM packs. In theory, one of the advantages of PCMs (beyond preventing freezing in a transport cold box) was that these could be cooled in the same refrigerator that is use for storing vaccines, and thereby, eliminating the need for health facilities to be equipped with a specific ice-pack freezer. Unfortunately, the risk of heat exposure to vaccines from cooling PCM packs in the same refrigerator is a concern and a separate refrigerator unit is required to store PCM packs. This adds to the cost of implementing this solution. The third main limitation concerns this analysis itself which is based on a relatively short time series of 6 months. A full 12 months of data would have provided sufficient data to capture seasonality effects, and confirm the trends of lowering the incidents of freezing in the vaccine cold chain.

Despite the limitations, the findings from this research have important policy and practice implications. For countries that are unable to purchase specialized vaccine cold chain, these findings highlight the benefits of switching to continuous temperature monitor practices as a way to safeguard the potency of vaccines in an end-to-end in-country vaccine supply chain, and as a managerial tool to ensure the continued effectiveness of national immunization programs worldwide.

## Figures and Tables

**Table 1:** Impact of the vaccine freeze-prevention solution on temperature metrics at each level.

	**No. of freeze alarms**		**% Temperature below 2** **°C**		**% Temperature between +2 °C to +8 °C**	
	Baseline	Demonstration	Baseline	Demonstration	Baseline	Demonstration
Regional level (x1)	0	0	0.5	2.3	96.3	91.7
District level (x12)	6	0	15.5	1.0	77.8	83.1
Health center (x25)	329	199	33.6	10.8	63.2	82.5
Storage (overall)	335	199	23.9	8.9	73.9	83.9
Transport (district to health centers)	na	na	13.8	1.7	59.9	65.7

## Footnotes

1 “*WHO/PQS Test Procedures E003/Guide 1.3: Failures at +32C and +43C tests (Day Night, holdover,stable-running).*

2 The paper is based on the current WHO Freeze alarm criteria (>60 consecutive min @ <−0.5 °C). −0.5 °C is the scientific freezing point of liquid vaccine. The actual freezing point is not a stable reference point although it is in most cases well below −0.5 °C. Unfortunately, because below −0.5 °C the vaccines freeze point is variable and unreliable, WHO has the policy to check vaccines that have been exposed with the shake test, but not to expose vaccines below the alarm standard for storage.

3 30-day temperature recorders meeting WHO/PQS Performance Specification E006/TR06.3: Models used in the Optimize study were: Storage – Log-tag TRED 30/7 with external temperature sensor. Transport – Log-tag TRID 30/7 with internal temperature sensor. Configuration of recorders in 2011 was not the same as in the 2012 baseline; in 2012 both the baseline and intervention recordings were made with identical configuration.

4 PCM are a group of chemicals that can prevent freezing since they freeze and melt at a temperature threshold of +4**˚**C/+5**˚**C . If frozen in a refrigerator set to maintain +2**˚**C, they solidify over-night. (Salthydrate C7) PCMs have only half the Latent heat capacity (140 kj/kgK) than water-ice (334 kj/kgK) so they keep cool for a shorter time.
